# Multigenic Natural Variation Underlies *Caenorhabditis elegans* Olfactory Preference for the Bacterial Pathogen *Serratia marcescens*

**DOI:** 10.1534/g3.113.008649

**Published:** 2013-12-17

**Authors:** Elizabeth E. Glater, Matthew V. Rockman, Cornelia I. Bargmann

**Affiliations:** * Department of Biology, Harvey Mudd College, Claremont, California 91711; † Department of Biology and Center for Genomics and Systems Biology, New York University, New York, New York 10003; ‡ Howard Hughes Medical Institute, Laboratory of Neural Circuits and Behavior, The Rockefeller University, New York, New York 10065

**Keywords:** *Caenorhabditis elegans*, olfaction, natural variation, *Serratia marcescens*

## Abstract

The nematode *Caenorhabditis elegans* can use olfaction to discriminate among different kinds of bacteria, its major food source. We asked how natural genetic variation contributes to choice behavior, focusing on differences in olfactory preference behavior between two wild-type *C. elegans* strains. The laboratory strain N2 strongly prefers the odor of *Serratia marcescens*, a soil bacterium that is pathogenic to *C. elegans*, to the odor of *Escherichia coli*, a commonly used laboratory food source. The divergent Hawaiian strain CB4856 has a weaker attraction to *Serratia* than the N2 strain, and this behavioral difference has a complex genetic basis. At least three quantitative trait loci (QTLs) from the CB4856 Hawaii strain (HW) with large effect sizes lead to reduced *Serratia* preference when introgressed into an N2 genetic background. These loci interact and have epistatic interactions with at least two antagonistic QTLs from HW that increase *Serratia* preference. The complex genetic architecture of this *C. elegans* trait is reminiscent of the architecture of mammalian metabolic and behavioral traits.

Individual differences in behavior have genetic and environmental components. The genetic basis of natural variation in behavior is generally understood to be complex, with multiple contributing loci that each explains only a fraction of the variance in a trait (Fisher 1918). Our current understanding of this variation is largely based either on association studies (such as GWAS), in which the effect of each locus is assessed across a variety of genetic backgrounds, or on studies that use recombinant inbred lines constructed from two parent strains, in which many segregating loci are examined in parallel (Altshuler *et al.* 2008; Lander and Botstein 1989). Both of these approaches screen broadly for variation, but the quantitative assumptions underlying their use are biased toward loci with additive effects that are insensitive to epistatic interactions or genetic background. In both cases, the effect size of each locus is averaged over all tested genetic backgrounds.

In experimental animals, defined genetic regions can be transferred between strains through introgression, holding the genetic background constant. This method has been historically important in immunological studies in mice, in which highly introgressed recombinant inbred lines defined specific immune functions within the major histocompatibility loci (Bach *et al.* 1972; McDevitt *et al.* 1972; McDevitt and Tyan 1968). Introgression is particularly valuable when multiple loci interact in unpredictable ways, as can occur in immune responses and metabolism-related traits (Bhatnagar *et al.* 2011).

*Caenorhabditis elegans* is an excellent model organism for studying natural variation. It reproduces as a self-fertilizing hermaphrodite with occasional male outcrossing, which facilitates the generation of isogenic strains compared to obligate sexual species. Different strains of *C. elegans* vary in a wide range of phenotypes, including foraging behavior, oxygen and carbon dioxide preference, susceptibility to pathogenic bacteria, and dauer development (Bendesky and Bargmann 2011; de Bono and Bargmann 1998; Hodgkin and Doniach 1997; McGrath *et al.* 2009; Persson *et al.* 2009; Schulenburg and Ewbank 2004; Schulenburg and Muller 2004; Viney *et al.* 2003). Loci affecting several of these traits have been identified through quantitative trait loci (QTLs) approaches, but the genetic architecture of many of these traits has not been fully examined. In this study, we examined the multigenic basis of a complex trait, bacterial preference behavior, using an introgression strategy.

*C. elegans* lives largely in association with human agriculture, where it feeds on a variety of bacteria and fungi associated with rotting fruit and plant matter (Felix and Duveau 2012). Among these microorganisms, the animal must choose food that is edible, nutritious, and nonpathogenic. Microbiomes are highly diverse, so *C. elegans* strains isolated from different regions may have adapted to local microbiota. In agreement with this possibility, different strains of *C. elegans* exhibit innate genetic variation in their interactions with specific bacteria. Wild-type *C. elegans* vary in their susceptibility to being killed by the bacterial pathogens *Bacillus thuringiensis* and *Serratia marcescens* (Schulenburg and Ewbank 2004; Schulenburg and Muller 2004), in their behavioral evasion of *Bacillus thuringiensis* (Schulenburg and Muller 2004), and in their ability to distinguish behaviorally among different species of bacteria (Volkers *et al.* 2013). In addition to exhibiting strong innate preferences, *C. elegans* can learn to avoid the odor of specific pathogenic bacteria after infection (Zhang *et al.* 2005) and will migrate away from toxic or inedible bacteria, in part based on olfactory cues (Melo and Ruvkun 2012; Pradel *et al.* 2007; Shtonda and Avery 2006). Both innate and learned odor responses are generated by a highly developed olfactory system with thousands of chemoreceptor genes (Bargmann 2006).

We examined the neuronal and genetic basis of *C. elegans* olfactory preference with a choice between the pathogenic bacteria *Serratia marcescens* and nonpathogenic *Escherichia coli* HB101. *S. marcescens* is highly attractive to and readily consumed by *C. elegans*, even though it establishes an intestinal infection that kills the worm after 2 to 3 d (Kurz *et al.* 2003). Although *C. elegans* is initially strongly attracted to a patch of *Serratia* bacteria, the worms will leave the bacteria after several hours through a learned avoidance mediated by the *tol-1* gene (Pujol *et al.* 2001). We examined natural variation in *Serratia* preference between the N2 Bristol laboratory strain and the CB4856 Hawaii strain (HW) using recombinant inbred lines, chromosome substitution strains, and introgression lines, and we found that multiple QTLs and multiple epistatic interactions influence olfactory preference behavior. The genetic complexity of this *C. elegans* trait recapitulates the genetic complexity of mammalian behaviors and suggests that introgression will be a valuable approach for finding underlying genes.

## Materials and Methods

### Nematode growth and strains

Strains were grown and maintained under standard conditions at 20**°** on nematode growth media (NGM) (Brenner 1974). L4 animals were placed on 100-mm NGM plates seeded with *E. coli* HB101 ATCC 33694 and their adult progeny were assayed 4 d later. A complete list of *C. elegans* strains is provided (Supporting Information, File S1).

### Bacterial strains

Bacterial strains were obtained from the American Type Culture Collection. Strains were *Serratia marcescens* ATCC 274 and *E. coli* HB101 ATCC 33694.

### Bacterial choice assay

The two-choice bacterial choice assay was modified from the work of Zhang *et al.* (2005). Briefly, bacteria grown overnight in LB at 26**°** were resuspended at an OD600 of 1.0 for *S. marcescens* or an OD600 of 10.0 for *E. coli* HB101, and 25 μl of each bacterial suspension was spotted onto an NGM plate and air-dried for 5 hr at 20**°**. At these OD600 values, both bacteria had approximately the same cellular density: at OD600 of 1.0, *S. marcescens* yields 2.1 × 10^9^ ± 1.5 × 10^9^ colony-forming units (cfu) per ml; at OD600 of 10, *E. coli* HB101 yields 3.2 × 10^9^ ± 1.7 × 10^9^ cfu per ml. Adult animals were washed three times in 1.5 ml S-basal buffer and 50–200 animals were placed with glass Pasteur pipette near the center of an NGM plate, equidistant from the two bacteria. Animals were allowed to move freely for 1 hr before being immobilized by 1 μl of 1 M sodium azide (movie of bacterial choice assay, File S2). We scored the number of animals on the *Serratia* lawn and the number of animals on the *E. coli* lawn. After 1 hr, less than 5% of animals were found outside the bacterial lawn for all strains tested; these animals were not counted. Assays for chromosome substitution strains and introgression strains were repeated at least five times on at least two different days. Assays for recombinant inbred advanced intercross lines (RIAILs) were repeated three to 10 times on at least two different days.

### Generation of introgression strains

Chromosome IV introgression strains were made by crossing N2 males to hermaphrodites from strain WE5239, which bears the CB4856 (HW) chromosome IV on an N2 background. The F2 progeny were screened for recombination events by PCR analysis of known chromosome IV polymorphisms between N2 and HW (www.wormbase.org) (Davis *et al.* 2005). F3 self-progeny homozygous for the recombinant chromosomes were identified by PCR genotyping, and homozygous strains were assayed in the bacterial choice assay in subsequent generations. Strains with a behavioral phenotype resembling the HW parent were then crossed to N2 males and the process was repeated to generate introgression strains containing smaller regions of HW DNA. Introgression strains were genotyped with SNPs identified in WormBase (www.wormbase.org). The genotypes of these lines can be found in File S4.

### Statistical analysis for determining QTLs

#### RIAIL analysis:

Seventy-two RIAILs, each genotyped at 1455 markers (for RIAIL genotypes, see Rockman and Kruglyak 2009), were phenotyped in three to 10 assays each, and the mean choice indices were analyzed by interval mapping in *R/qtl* (Broman *et al.* 2003) after they were Box-Cox–transformed to approximate normality (Venables and Ripley 2002). The genome-wide *P* of the peak LOD score was estimated by 1000 permutations (Churchill and Doerge 1994). Qualitatively identical results were found with nonparametric interval mapping.

To directly evaluate a contribution from the introgression line-defined QTLs, we used the fitqtl function of *R/qtl*, which performs an ANOVA to test the significance and to estimate the variance explained for specified QTL in a multiple QTL model. This has the advantage of using imputed genotypes or genotype probabilities at QTL rather than relying on marker class means.

#### Introgression line analysis and common segment method:

The choice index of each introgression line (47 lines), or a subset of these lines, was compared to the choice index of N2 using ANOVA with Dunnett correction for multiple comparisons (*P* < 0.05). Strains with phenotypes that differ significantly from the N2 are likely to contain one or more QTLs. Strains that do not differ significantly from N2 likely do not contain a QTL or contain a QTL and an additional suppressor. We attempted to explain the phenotypes of each strain that differs from N2 by invoking the fewest necessary QTLs (*i.e.*, common segments, shared by strains) (Shao *et al.* 2008). Strains that contain those QTLs but are not different from N2 are inferred to carry suppressors, whose number we minimize in the same manner. The suppressors are invoked by parsimony and are not subjected to formal hypothesis test (Shao *et al.* 2010).

#### Introgression line analysis and sequential minimum spanning tree method:

In the sequential method (Shao *et al.* 2010), strains are compared two at a time and significant differences imply that the chromosome segments not shared by the two strains harbor a QTL. To minimize the number of strain comparisons and to maximize localization resolution, the method compares pairs of strains that are most genetically similar to one another. The sequence of comparisons is determined by constructing a minimum spanning tree that connects the strains according to their pairwise similarity. In our implementation, we calculated genetic similarity by estimating the number of base pairs that differ between each strain, assuming that breakpoints are at the midpoints of marker intervals. We used the spantree function of the R package vegan (Oksanen *et al.* 2012) to find a minimum spanning tree and we tested for phenotypic differences between pairs of strains adjacent on the tree by *t* test with Bonferroni correction.

## Results

### Wild-type strains vary in bacterial preference

**Figure 1:**
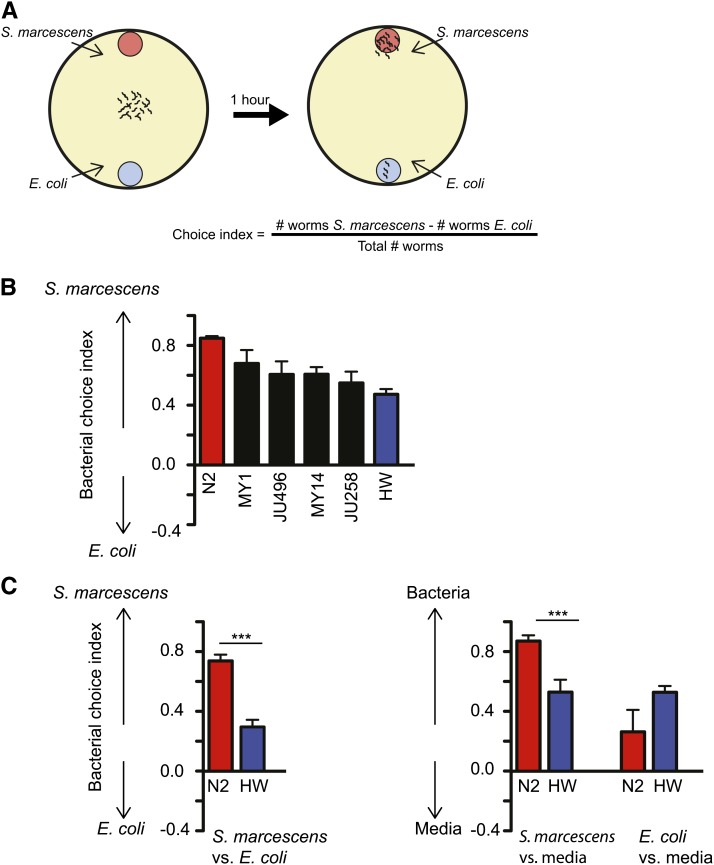
Bacterial choice behavior varies among wild-type *C. elegans* strains. (A) Cartoon of the bacterial choice assay. Approximately 100 worms are placed on the agar plate between two patches of bacteria, which they can approach by olfactory chemotaxis. (B) Bacterial choice index of six wild strains. HW, strain CB4856. n ≥ 6 assays. (C) Choice assays for *Serratia vs. E. coli*, *Serratia vs.* LB media, and *E. coli vs.* LB media conducted in parallel experiments. ***P < 0.001, *t* test, n ≥ 6 assays. S.E.M. represented by error bars.

Bacterial preferences of *C. elegans* were evaluated using a bacterial choice assay in which worms migrate to one of two patches of bacteria on opposite sides of an agar plate (Figure 1A) (Zhang *et al.* 2005). The first approach of the animals over 1–2 hr is dominated by their olfactory preferences for volatile odors released by the bacteria. We examined two strains, *S. marcescens* ATCC 274 and *E. coli* HB101. Surprisingly, although *S. marcescens* is a bacterial pathogen that can kill *C. elegans*, it was more attractive to the wild-type *C. elegans* strain N2 than its standard laboratory food source, *E. coli* (Figure 1B).

An animal’s preference for different food sources should vary based on its natural ecology, and recent studies of *C. elegans* indicate that it is found in human-associated environments with a variety of different bacteria (Felix and Duveau 2012). We examined bacterial preference behavior in wild-type strains isolated from different environments and found that wild-type strains of *C. elegans* varied in their preference between *S. marcescens* ATCC 274 and *E. coli* HB101 (Figure 1B). Among six tested strains, the N2 laboratory strain had the strongest preference for *Serratia* over *E. coli*, and a highly divergent strain, HW, had the weakest preference for *Serratia*.

In a choice between bacteria and the bacterial growth media alone (LB), N2 had a significantly stronger preference for *Serratia* than HW (Figure 1C). A trend toward an increased HW preference for *E. coli* over media was not statistically significant (Figure 1C). These results indicate that the response to *Serratia* is the main source of genetic variability between the N2 and HW strains, although the *E. coli* response may also contribute to their distinct preferences.

### Segregation of preference behavior in recombinant inbred lines

**Figure 2:**
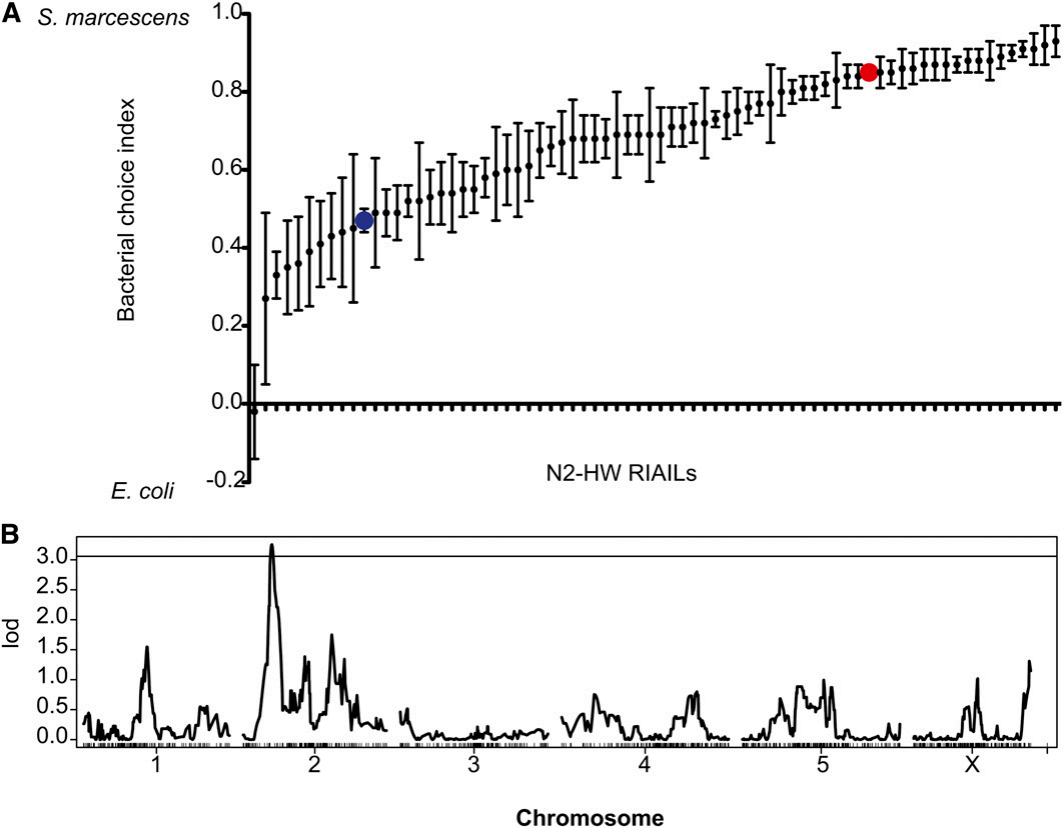
QTL mapping of bacterial choice index with N2–HW recombinant inbred advanced intercross lines (RIAILs). (A) Bacterial choice index of 72 N2–HW RIAILS (black), N2 (red), and HW (blue). RIAIL strain names and choice index are in File S3. (B) Logarithm of odds (LOD scores) along chromosomes for RIAILs shown, with horizontal line denoting threshold for genome-wide significance (*P* = 0.05).

To determine the genetic basis of natural variation in bacteria preferences between N2 and CB4856 (HW), we first assayed 72 genotyped N2–CB4856 RIAILs (Rockman and Kruglyak 2009) in the bacterial choice assay (Figure 2A). These strains have been genotyped at more than 1000 informative loci and have been used successfully to identify loci affecting a variety of behavioral, developmental, and life history traits (Bendesky *et al.* 2011; Gaertner *et al.* 2012; McGrath *et al.* 2009; Palopoli *et al.* 2008; Seidel *et al.* 2008). Variance among RIAIL strains accounted for 46.3% of the total variance in bacterial preference across assays, providing an estimate of broad-sense heritability of the trait (F_71,408_ = 4.95; *P* < 10^−15^). The RIAILs varied smoothly in their bacterial preference, suggesting that more than one gene affects bacterial choice (Figure 2A). In addition, several strains had bacterial preference more extreme than either starting strain, a pattern of transgressive segregation suggesting that N2 and HW each carry alleles that act in both directions, possibly in background-dependent manners (Figure 2A). Although this pattern suggests that there are multiple segregating loci in the strains, linkage analysis of the RIAILs yielded only a single QTL on chromosome II at genome-wide significance (II:2808858 with LOD = 3.255; genome-wide *P* = 0.036) (Figure 2B). The HW allele at this QTL decreases behavioral preference for *Serratia* bacteria.

### Multiple loci that differ between N2 and HW affect bacterial preference

**Figure 3:**
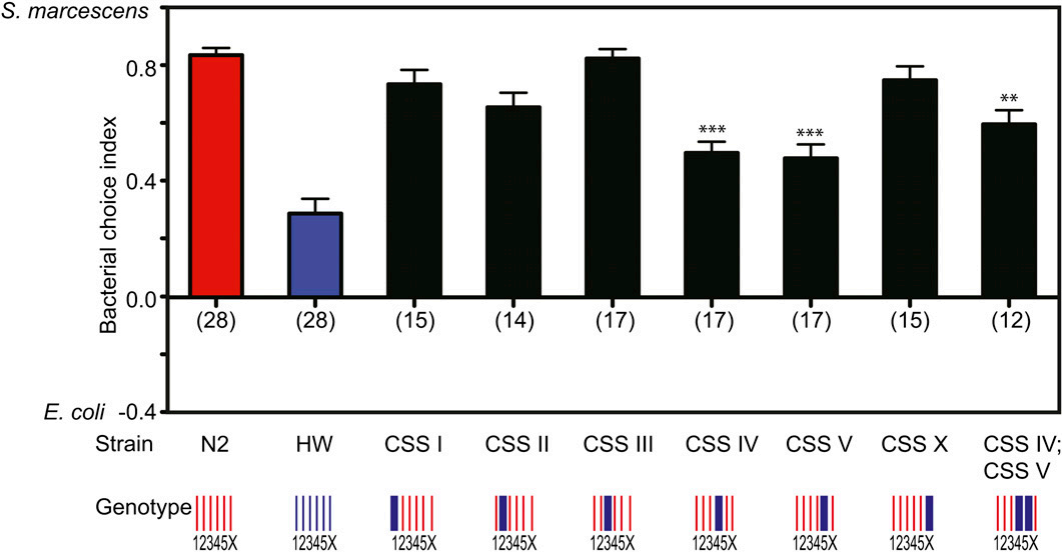
Quantitative trait loci on two chromosomes underlie natural variation in bacterial choice behavior. Bacterial choice behavior of chromosome substitution strains. “Genotype” shows chromosomes (blue denotes HW DNA; red denotes N2 DNA). ***P < 0.001, **P < 0.01 compared to N2 by ANOVA with Dunnett test, n in parentheses under each bar. CSSIV, CSSV, and CSSIV; CSSV were statistically indistinguishable by *t* test. S.E.M. represented by error bars.

To further examine the significance of the QTLs on chromosome II, and to probe the genetic structure of the bacterial preference trait more generally, we next assayed bacterial preference in six N2–HW chromosome substitution strains (CSSs) in which a single homozygous HW chromosome replaced the corresponding chromosome in an otherwise N2 background (Glauser *et al.* 2011). The strain bearing HW chromosome II (CSSII) closely resembled N2, showing no evidence of HW-like bacterial preference (Figure 3). This result could indicate that the marginally significant QTLs identified using the RIAILs were false-positive, or that the CSSs were false-negative. We tested the bacterial choice behavior of an introgression strain with the HW chromosome II QTL predicted by RIAIL analysis and found that the strain had an N2-like phenotype (Figure S1). This strain may have an N2-like phenotype because this region does not contain a QTL or because this region interacts epistatically with other QTL to generate a HW-like phenotype. The latter interpretation would be consistent with a complex genetic architecture for bacterial preference, as suggested by transgressive segregation in the RIAILs.

Examining the other CSS strains provided support for a complex genetic architecture. Strains bearing either HW chromosome IV (CSSIV) or chromosome V (CSSV) had a HW-like phenotype, whereas all other CSS lines had an N2-like phenotype (Figure 3). These results suggest that at least two regions, one each on chromosomes IV and V, contain QTLs for bacterial choice behavior, although neither emerged from the RIAILs.

To assess the interaction between these two chromosomes, we generated a CSS with both chromosome IV and chromosome V from the HW background. The behavior of this strain was statistically indistinguishable from either individual CSS (Figure 3). Therefore, both chromosome IV and chromosome V bear QTLs that affect bacterial preference, but these QTLs are not additive.

### Mapping QTL on chromosome IV

**Figure 4:**
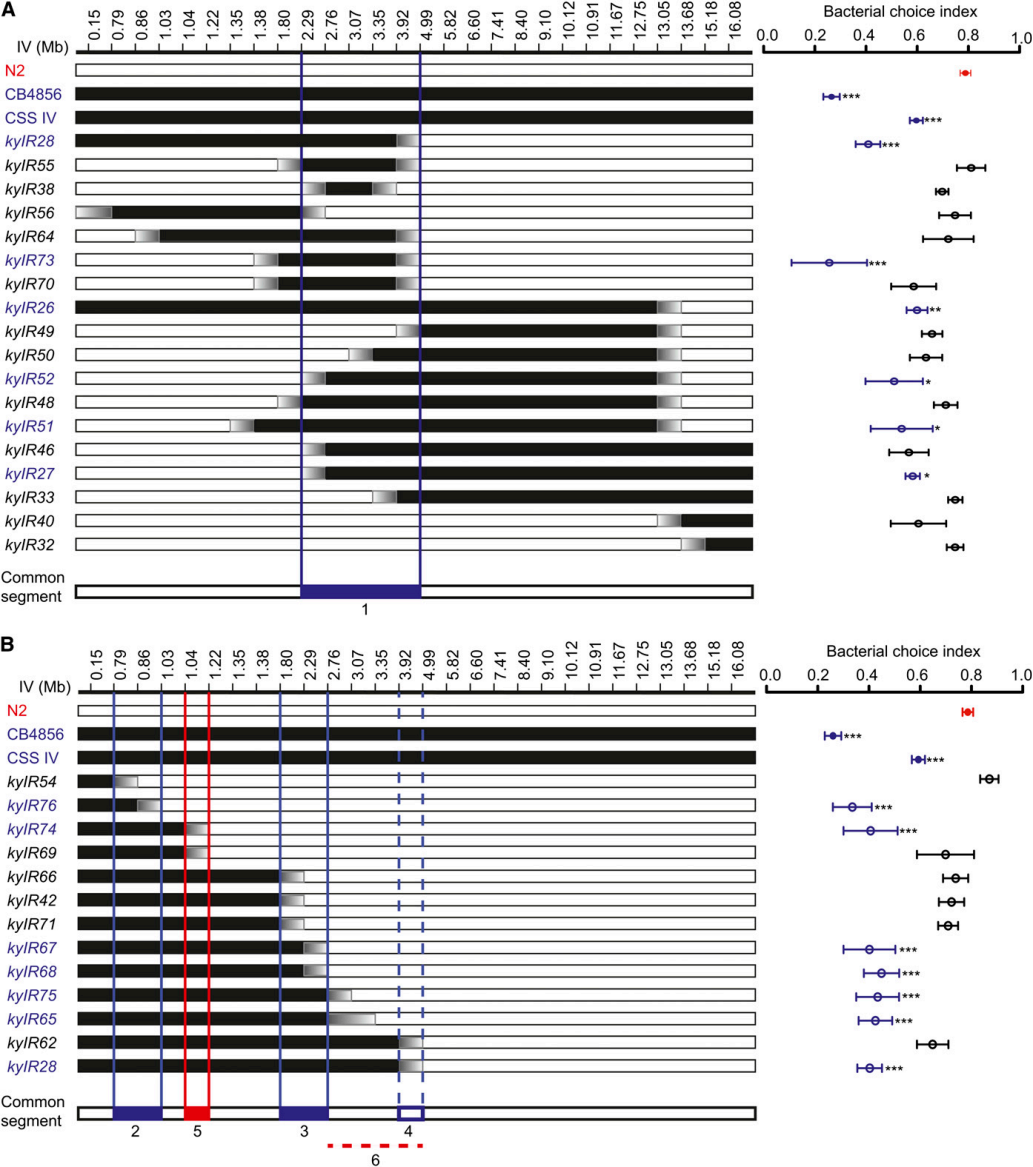

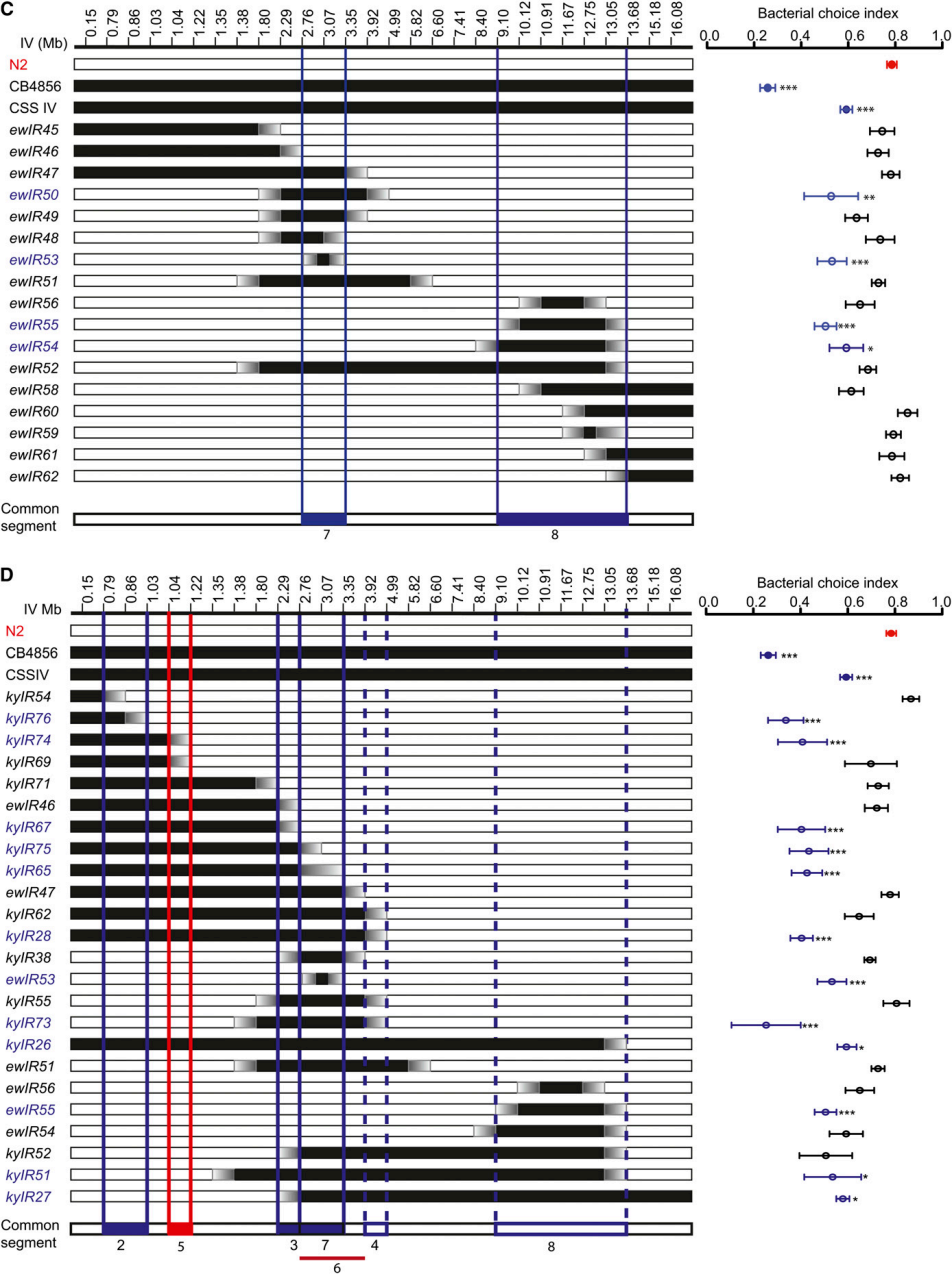
Multiple QTLs on chromosome IV. QTLs for bacterial choice of introgression lines derived from CSSIV (this article) and from recombinant inbred lines (Doroszuk *et al.* 2009). Left, Genotypes are shown for various genetic markers (white is N2; black is HW; graded from white to black indicates unknown genomic regions between genotyped SNPs. Right, Bacterial choice behavior of introgression lines. Blue markers indicate lines that differ significantly from N2. ***P < 0.001, **P < 0.01, *P < 0.05, ANOVA with Dunnett, n ≥ 5 assays. S.E.M. represented by error bars. Chromosome IV introgression line strain names, choice index, and genotype at additional genetic markers are in File S4. Introgression lines were analyzed by the common segment method to determine QTLs. The inferred locations of QTLs that decrease *Serratia* preference are indicated by blue vertical lines on the genetic map; antagonistic QTLs that restore N2-like phenotype are indicated by red vertical lines on the genetic map. N2 and CB4856 were tested as well as subset of introgression strains on each day. The N2 and CB4856 data shown are the average data for N2 and CB4856 tested on all days that the introgression strains were tested. (A) Initial set of lines derived from CSSIV. Using the common segment method, recombinant strains that differed significantly from N2 shared an HW region (∼2.29 to ∼4.99 MB; QTL1). (B) Introgression lines that begin at the left telomere of chromosome IV and are derived from *kyIR28*. Common segment method reveals QTL2 and QTL3 that confer HW-like behavior and antagonistic QTL5 that confers N2-like behavior. QTL4 and antagonistic QTL6 are supported by only one line (*kyIR62*) and are indicated with dashed lines. (C) Independent introgression lines (*ewIR*) from study by Doroszuk *et al.* (2009). Common segment method identifies QTL7 and QTL8. (D) Analysis of all chromosome IV introgression lines (*kyIR* and *ewIR*). All lines were analyzed, but only a subset that includes all lines significantly different from N2 is shown. Analysis of all lines yielded the same QTLs as in subsets, with three exceptions: QTL3 is a smaller region because it is defined by both *ewIR* and *kyIR* lines (specifically *ewIR46* and *kyIR67*); antagonistic QTL6 is defined by two lines instead of one (*ewIR47* and *kyIR62*); and QTL8 is only supported by one line, *ewIR55*. The line *ewIR54* is no longer significantly different from N2 when part of a larger data set. Complete explanation of chromosome IV QTLs defined by common segment method appears in File S6.

Regions of chromosome IV that affected bacterial preference were defined further by making recombinants between CSSIV and N2. Recombinants resulting from two or three iterative rounds of recombination [(CSSIV × N2) × N2 (× N2)] were genotyped across chromosome IV and tested for preference behavior as homozygotes. All recombinant strains that differed significantly from N2 shared an HW region between 2.29 and 4.99 MB, suggesting the presence of a QTL conferring HW-like behavior in this region (QTL1) (Figure 4A). However, these introgression lines provided relatively little power to resolve QTLs, because the HW-derived DNA segments were large and contained relatively few breakpoints.

The introgression strain *kyIR28* resembled CSSIV in the choice index but contained only 5 MB of HW DNA beginning at the left telomere of chromosome IV (Figure 4A). Using *kyIR28* as a starting point, we generated additional recombinants as a nested set of introgression lines that derived from *kyIR28* and included HW sequences beginning at the left telomere of chromosome IV. These strains were tested for preference behavior as homozygotes (Figure 4B). Direct inspection of their phenotypes suggested that *kyIR28* probably contains more than one QTL: two groups of strains within the nested series were HW-like (*kyIR76,74* and *kyIR67,68,75,65*), but another group of nested strains were N2-like *(kyIR69,66,42,71*). The simplest explanation for these results is the existence of two QTLs that confer HW-like behavior (QTL2 and QTL3), separated by a third antagonistic QTL from the HW strain that confers N2-like behavior (QTL5). Statistical testing of these strains using the “common segment” method as described by Shao *et al.* (2010) using ANOVA with Dunnett correction for multiple testing supported the existence of each of these three QTLs (*P* < 0.05) (Figure 4B).

Statistical testing also provided support for two additional QTLs of opposite signs, one conferring HW-like behavior (QTL4) and one conferring N2-like behavior (QTL6). The existence of QTL4 and QTL6 was supported only by a single introgression strain, *kyIR62*, whereas the existence of QTL2, QTL3, and QTL5 were all supported by multiple strains (Figure 4A).

The antagonistic interactions among QTLs in these strains suggest that HW QTL do not uniformly promote HW-like behavior; some regions of HW DNA, including QTL5 and possibly QTL6, favor N2-like behavior.

### Chromosome IV QTL defined by independent introgression lines

It has been suggested that the most powerful way to identify multiple QTLs is to use contiguous congenic strains that tile a chromosome in small segments with minimal overlap (Rapp and Joe 2012). In *C. elegans*, congenic strains of this design that span the genome have been generated between the N2 and HW strains and colleagues (Doroszuk *et al.* 2009). We systematically examined the strains that covered chromosome IV to test the power of these strains for identifying QTLs and to ask if congenic strains generated by different approaches would yield similar QTLs.

Two QTLs that confer HW-like behavior were identified from this analysis (Figure 4C). One, QTL7, fell in the same region as QTL1. The second, QTL8, fell on the right arm of the chromosome, in a region that was poorly resolved by breakpoints in the previous set of introgression lines (Figure 4A) but was well-resolved in this set (Figure 4C).

Combining all data from all introgression lines into a single dataset yielded results consistent with those from individual strains (Figure 4D), with four to five QTLs favoring HW-like behavior (QTL2, QTL3, QTL4, QTL7, QTL8) and two antagonistic QTLs favoring N2-like behavior (QTL5, QTL6). Contrary to the simple expectation that chromosome IV might have one major locus for bacterial preference, the introgression lines defined multiple QTLs, whose numbers increased as the number of informative recombination breakpoints increased.

**Figure 5:**
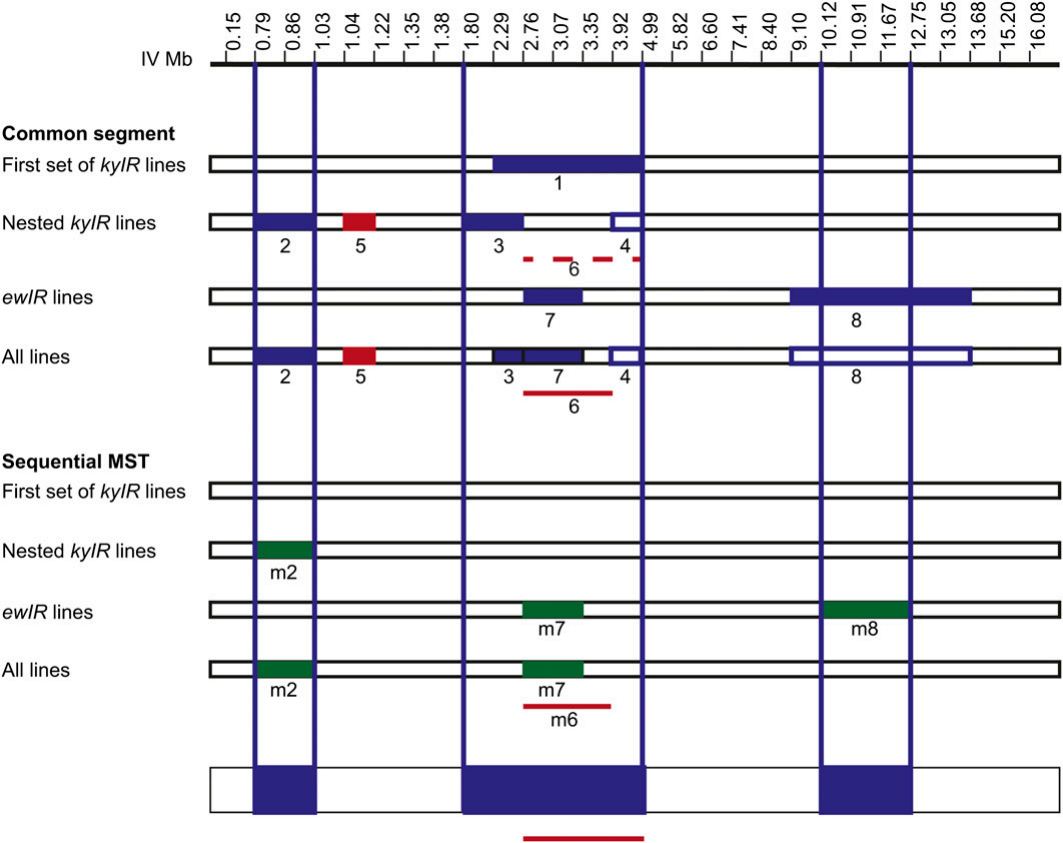
Summary of QTLs on chromosome IV. Location of QTLs determined by common segment method (Figure 4) [blue and red (antagonistic QTLs)] and by sequential minimum spanning tree method [green and red (antagonistic QTLs)] showing generally similar locations of QTL (bottom of figure). In the sequential minimum spanning tree method, the introgression lines that differ significantly from each other (*t* test with Bonferonni correction, *P* < 0.05) in both subsets of lines and all lines were as follows: *kyIR76* and *kyIR54*, defining QTL m2 (0.79–1.03 Mb) and *ewIR53* and *N2*, defining QTL m7 (2.76–3.35 Mb). The difference between *ewIR58* and *ewIR60*, defining QTL m8 (10.12–12.75 Mb), was significant in *ewIR* lines, but not all lines combined. The significant difference between *ewIR47* and *kyIR65*, defining antagonistic QTL m6 (∼2.76 to ∼3.92 Mb), was present only in all lines combined. Complete explanation of chromosome IV QTL defined by sequential MST appears in File S7.

The common segment method has a long history of use in congenic inbred strains (Snell and Bunker 1965), but alternative methods for mapping have recently been proposed to be more rigorous. We used the sequential minimum spanning tree method (Shao *et al.* 2010) to examine the same set of introgression lines characterized and found that this method identified four QTLs: QTL m2, which overlapped with QTL2; QTL m7, which overlapped with QTL7; antagonistic QTL m6, which overlapped with QTL6; and QTL m8, identified only in the *ewIR* set, which overlapped with QTL8 (Figure 5). The sequential method uses a very stringent Bonferroni correction for multiple testing; less stringent approaches (*e.g.*, false discovery rate) suggest the presence of multiple additional QTLs coincident with those found by the common segment method.

### Initial localization of chromosome V QTL

**Figure 6:**
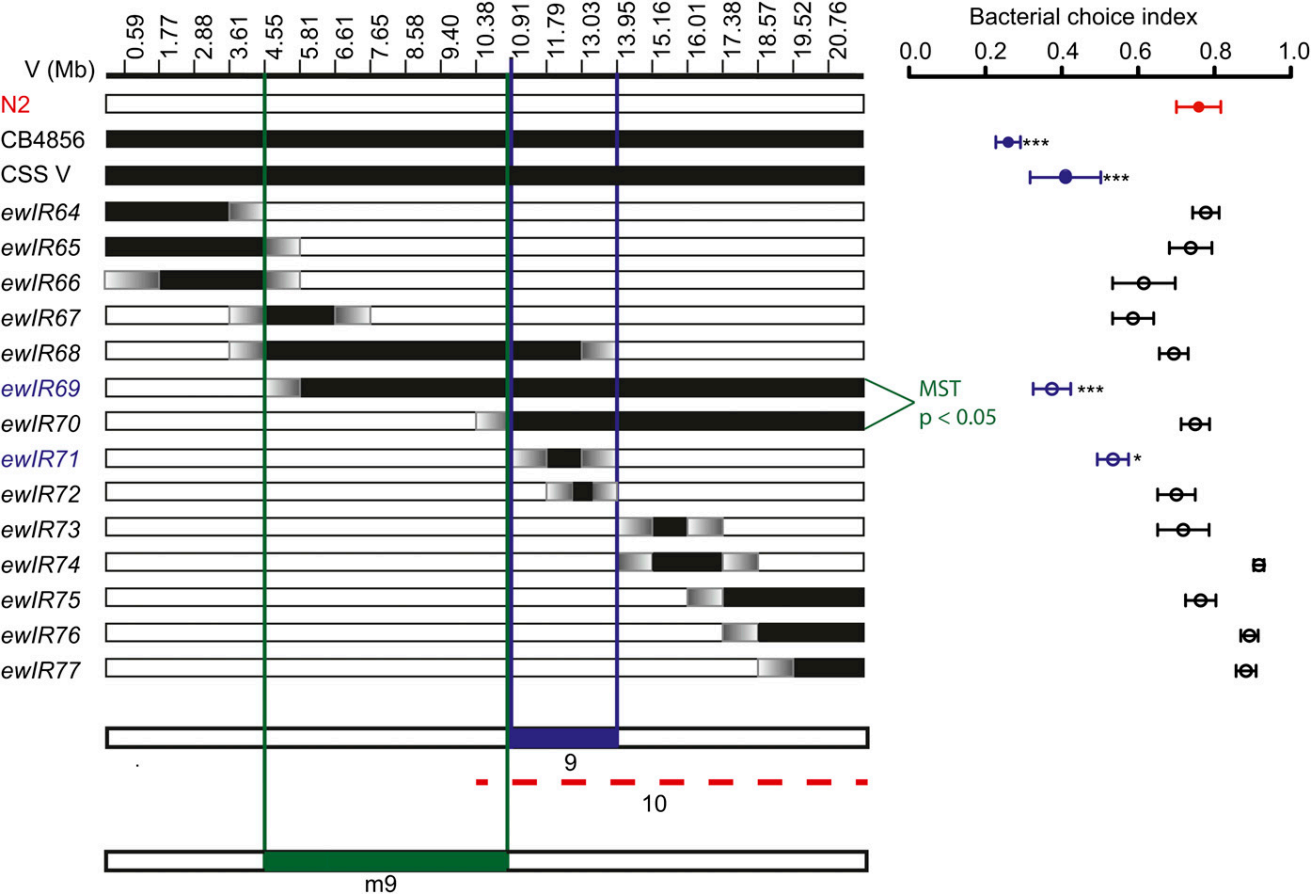
At least one quantitative trait locus on chromosome V. QTLs for bacterial choice of introgression lines derived from recombinant inbred lines (Doroszuk *et al.* 2009). Data are portrayed as in Figure 4. Chromosome V introgression line strain names and choice index appear in File S5. The common segment analysis of chromosome V identified QTL9 (∼10.91 to ∼13.95), defined by the introgression line *ewIR71* that differed significantly from N2. Antagonistic QTL10 is defined by the strain *ewIR70 that* includes QTL9 and had an N2-like behavioral preference. The sequential minimum spanning tree method identified QTL m9 because of the significant difference in the choice index between *ewIR69* and *ewIR70*. Additional explanation of chromosome V QTL defined by the common segment method and sequential MST appears in File S8.

To further characterize the inferred QTL or QTLs on chromosome V suggested by the CSSV strain (Figure 3), we examined minimally overlapping congenic strains generated between the N2 and HW strains (Doroszuk *et al.* 2009). The common segment analysis assuming the smallest possible number of contributing QTLs on chromosome V identified QTL9 (∼10.91 to ∼13.95), defined by the introgression line *ewIR71* that differed significantly from N2 (Figure 6). However, the strain *ewIR70* that included this region and additional sequences had N2-like behavioral preference, suggesting that one or more antagonistic QTLs on chromosome V modify the QTL9 preference. The sequential minimum spanning tree method also identified one QTL (QTL m9) in an interval adjacent to QTL9.

The full set of suggested QTLs for bacterial preference converged on several similar regions for chromosome IV but were less well-defined for chromosome V (Figure 5 and Figure 6). Together, our mapping data suggest that there are at least four and probably five or six QTLs on chromosome IV and chromosome V that confer HW-like behavior in the HW strain, along with at least two antagonistic loci.

**Figure 7:**
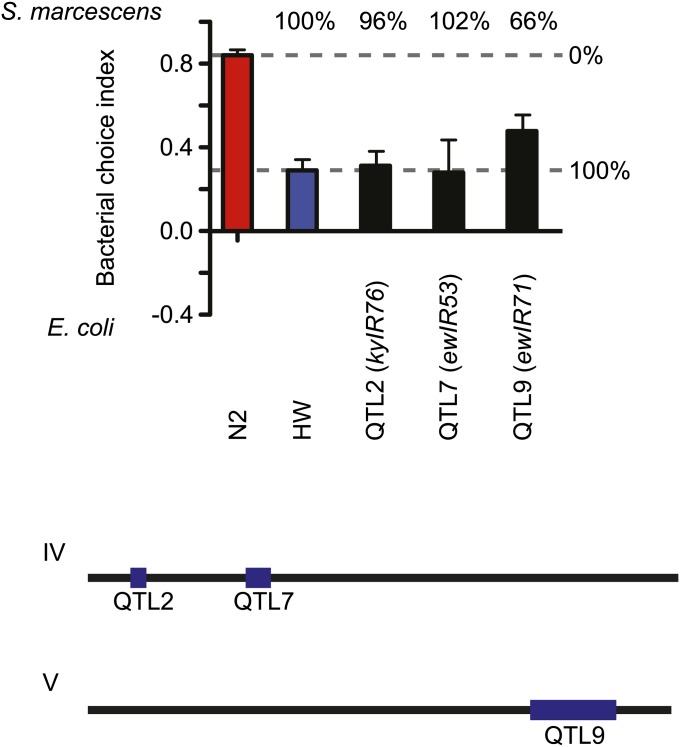
Effect sizes of individual QTL on an N2 background. Introgression strains that should each contain a unique QTL (or set of QTLs). Two loci are on chromosome IV: QTL2 (*kyIR76*), ∼0.79 to ∼1.03 Mb, and QTL7 (*ewIR53*), ∼2.76 to ∼3.35 Mb. One locus is on chromosome V: QTL9 (*ewIR71*), ∼10.91 to ∼13.95 Mb. Each QTL was backcrossed two additional times onto the N2 background before testing, yielding choice indexes slightly different from the original strains in Figure 4. Percentages above bars indicate the preference behavior of each strain as a percentage of the preference difference between N2 and HW. Horizontal dashed lines indicate choice index for 0% and 100% preference difference between N2 and HW. S.E.M. represented as error bars. At bottom, blue segments indicate approximate location of QTLs.

To estimate the effect size of individual QTLs, we examined the behavior of introgression strains that should each contain a unique QTL among those defined here, after further backcrossing onto a common N2-like genetic background. This analysis was possible for QTL2, QTL7, and QTL9, defined by the nonoverlapping introgression lines *kyIR76*, *ewIR53*, and *ewIR71*, respectively. The preference behavior in each strain was strongly HW-like, ranging from 66% to 102% of the preference difference between N2 and HW (Figure 7). The cumulative phenotypic effect of these three QTLs was 264%, exceeding the 100% starting difference between the two parental strains.

## Discussion

### Preference for *Serratia* bacteria

Animals from the HW had a lower preference for *Serratia* than N2 animals, and four other wild strains had intermediate preferences compared to these two strains. It is surprising that *C. elegans* has a strong preference for *S. marcescens*, a pathogenic bacteria that can kill infected animals in a few days (Mallo *et al.* 2002). This may be an example of a host–pathogen evolutionary arms race in which the pathogen is winning by attracting its host (Niu *et al.* 2010) or a fortuitous event in which *Serratia* odors resemble those of other harmless bacteria. Although there should be a strong selection for avoidance of this odor, the level of complexity of the microbiome may challenge even the considerable genetic capacity of *C. elegans* for chemosensation.

### Complex genetics of bacterial preference traits

The analysis of N2–HW strains suggests the existence of as many as nine QTLs on HW chromosomes IV and V and perhaps one on chromosome II that affect bacterial preference. The location and number of QTLs identified were sensitive to the exact strains and analysis methods that were used, but several different approaches and two independently derived sets of introgressed strains yielded similar locations for most QTLs on chromosome IV (Figure 5). QTL alleles on two different HW chromosomes favor HW-like behavioral preferences, whereas additional QTLs have antagonistic effects.

Identifying QTLs for bacterial preference traits proved unexpectedly challenging, but it became more straightforward as smaller regions of HW DNA were successively introgressed onto an N2 background. The RIAILs with a 50:50 mix of HW and N2 DNA yielded the fewest QTLs (Figure 2), the chromosome substitution strains were more informative (Figure 3), and the smallest nested introgression strains (Figure 4B) gave the most informative and interpretable results. These results are most simply explained by the relatively complex genetic architecture of the underlying trait and particularly the presence of alleles in the HW strain that suppress the effects of HW alleles at other loci. Our results suggest that this complex trait is most effectively dissected by analyzing small genetic regions in a common strain background, with the knowledge that this approach (and probably any experimentally feasible approach) will reveal only a subset of the QTLs.

Although the bacterial chemotaxis assay uses a scale that is, in principle, able to detect additive factors, the QTLs on chromosomes IV and V appeared not to have additive effects on preference (Figure 3). This observation suggests that epistatic interactions among QTLs affect the behavioral preference phenotype, as is seen in many other systems. For example, natural variation in aggressive behavior between two wild-type strains of *Drosophila* involves at least five QTLs with epistatic interactions (Edwards *et al.* 2009). In *C. elegans*, epistatic interactions among multiple loci that vary between N2 and HW strains cause synergistic effects on thermal preference (Gaertner *et al.* 2012). Multiple epistatic QTLs contribute to natural variation in metabolic, blood, and bone trait differences between two wild-type strains of mice (Shao *et al.* 2008). Discovery of the mouse metabolic QTL was facilitated by characterizing nested introgression lines within a chromosome (Shao *et al.* 2008), the approach that also succeeded best in our study of *C. elegans* preference behavior.

Although several groups have successfully identified N2–HW QTLs using recombinant inbred advanced intercross lines, the analysis of RIAILs did not identify any of the QTLs for behavioral preference defined by the introgression approach, despite having sufficient power to detect QTLs that explain 30% of variance among RIAILs (Figure S2). We confirmed this negative result in the RIAIL lines with a multiple QTL model that includes the QTLs defined in the introgression lines; none of the QTLs defined by introgression lines was significant in any model incorporating some or all of them with or without interactions. Given the apparent prevalence of epistasis among QTLs, this discrepancy is explained by the large number of segregating genotypes compared to the number of tested RIAILs. Only 72 RIAILs were tested, a number that is small relative to the 128 possible genotypes at seven QTLs. With this genetic complexity, even QTLs that individually had large effects on specific backgrounds became undetectable when averaged across backgrounds. These results point out the value of testing smaller, defined genomic regions in introgression lines as a complementary approach to combining loci randomly in conventional RIL analysis.

### Similarities between mammalian and *C. elegans* complex trait genetics

This introgression analysis of *C. elegans* odor preference yielded results strikingly similar to an analysis of mouse metabolic and behavioral traits from 22 introgression lines with BALB/c chromosomes introduced into the C57B6 strain (Shao *et al.* 2008). First, the mouse chromosome substitution strains showed that for many traits, the effect size of a single chromosome was at least half of the total difference between the two starting strains. Second, many chromosome substitutions could affect any single metabolic or behavioral trait, so that the total effect sizes added together often represented 600% or more of the difference between the two starting mouse strains. Third, adding together multiple chromosome substitutions did not result in additive effects on the traits, and extensive epistasis often masked or reversed the effect of single chromosomes.

Population genetic analysis is appropriately focused on trait variance; however, from a mechanistic perspective, it is more straightforward to examine each genetic variant in a defined background before reconstructing the entire system. Therefore, applying introgression studies to define genetic factors may be a valuable approach to problems in behavior, metabolism, and other complex traits.

### Food choice behavior evolves rapidly

Increasing evidence suggests that taste and olfactory preferences are particularly fast-evolving behaviors that coordinate the behavioral and metabolic specializations of a species. For example, over the past 500,000 years, *Drosophila sechellia* has acquired metabolic specializations for growth on the toxic morinda fruit, in tandem with olfactory preferences for the same fruit (Jones 1998; McBride 2007; Stensmyr 2009). Over several million years, felines with a carnivorous diet that lacks sugar have accumulated inactivating mutations in the *Tas1r3* receptor gene, which is required for sweet taste in other mammals (Li *et al.* 2005). Strong recent signatures of positive selection on human bitter taste receptor genes suggest that dietary pressures, such as recognizing toxic foods, may also have left their mark on human sensory preference (Campbell *et al.* 2012; Li *et al.* 2011).

The chemosensory system of *C. elegans* is rapidly evolving compared to the rest of its genome, suggesting that it is under positive selection (Robertson 1998, Robertson 2000; Stewart *et al.* 2005; Thomas *et al.* 2005). Nearly 2000 *C. elegans* genes encode G-protein-coupled chemoreceptor genes, representing 5% to 10% of all protein-coding genes, and these genes are divergent among *C. elegans* wild isolates and among *Caenorhabditis* species (Stewart *et al.* 2005; Thomas and Robertson 2008). We speculate that the spectrum of wholesome and pathogenic bacteria in different environments generates local selective pressures on chemoreceptors and other *C. elegans* genes and subsequent within-species genetic diversity. This suggestion is consistent with the cosmopolitan lifestyle of *C. elegans* and its broad dispersion through a variety of human agricultural environments.

## Supplementary Material

Supporting Information
