# Supplementary material for: Multigenic Natural Variation Underlies Caenorhabditis elegans Olfactory Preference for the Bacterial Pathogen Serratia marcescens
Source: G3 (Bethesda). 2013 Dec 17;4(2):265–76. doi: 10.1534/g3.113.008649 (PMC3931561; doi:10.1534/g3.113.008649)
Supplement: Supporting Information [file supp_4_2_265__index.html]

Multigenic Natural Variation Underlies Caenorhabditis elegans Olfactory Preference for the Bacterial Pathogen Serratia marcescens — Supporting Information 

# Multigenic Natural Variation Underlies *Caenorhabditis elegans* Olfactory Preference for the Bacterial Pathogen *Serratia marcescens*

## Supporting Information for Glater, Rockman, and Bargmann, 2014

**Files in this Data Supplement:**

- Supporting Information - Files S1-S8 and Figures S1-S2 (PDF, 629 KB)
- Figure S1 - Bacterial choice behavior of introgression strain with chromosome II QTL predicted by RIAIL analysis. (PDF, 442 KB)
- Figure S2 - Power analysis of RIAILs. (PDF, 287 KB)
- File S1 - Supplemental Methods (PDF, 524 KB)
- File S6 - Additional explanation of Chromosome IV QTLs determined by common segment method. (PDF, 522 KB)
- File S7 - Explanation of Chromosome IV QTLs defined by sequential minimum spanning tree method. (PDF, 519 KB)
- File S8 - Explanation of Chromosome V QTLs by common segment and sequential minimum spanning tree methods. (PDF, 406 KB)
- File S4 - Chromosome IV Introgression lines, bacterial choice phenotypes and genotypes (.xlsx, 62 KB)
- File S5 - Chromosome V Introgression lines, bacterial choice phenotypes and genotypes (.xlsx, 34 KB)
- File S2 - Movie of N2 worms in bacterial choice assay (.wmv, 1 MB)
- File S3 - RIAIL bacterial choice phenotypes (.xls, 45 KB)
