# Supplementary material for: Multigenic Natural Variation Underlies Caenorhabditis elegans Olfactory Preference for the Bacterial Pathogen Serratia marcescens
Source: G3 (Bethesda). 2013 Dec 17;4(2):265–76. doi: 10.1534/g3.113.008649 (PMC3931561; doi:10.1534/g3.113.008649)
Supplement: Supporting Information [file supp_g3.113.008649_008649SI.pdf]

G3 · Genes | Genomes | Genetics

Multigenic natural variation underlies *Caenorhabditis elegans* olfactory preference for the bacterial pathogen *Serratia marcescens*

Elizabeth E. Glater*[1], Matthew V. Rockman[§], and Cornelia I. Bargmann[†]

[*]Department of Biology, Harvey Mudd College, Claremont, CA 91711
[§]Department of Biology and Center for Genomics and Systems Biology, New York University, New York, NY 10003
[†]Howard Hughes Medical Institute, Laboratory of Neural Circuits and Behavior, The Rockefeller University, New York, NY 10065

[1]Corresponding author: Department of Biology, Harvey Mudd College, Claremont, CA 91711. E-mail: Elizabeth_Glater@hmc.edu

DOI: 10.1534/g3.113.008649

File S1

Supplemental Methods

***C. elegans* Strains**

<u>'Wild-type' strains</u>

Strain	*Origin*
N2	Bristol, England (FATT and DOUGHERTY 1963)
CB4856	Hawaii, USA (described in (HODGKIN and DONIACH 1997))
MY1	Lingen, Germany (HABER *et al.* 2005)
MY14	Mecklenbeck, Germany (HABER *et al.* 2005)
JU258	Madeira, Portugal (BARRIERE and FELIX 2005)

<u>Chromosome substitution strains</u>

WE5236 (CSSI), WE5237 (CSSII), WE5238 (CSSIII), WE5239 (CSSIV), WE5240 (CSSV), WE5241 (CSSX) (GLAUSER *et al.* 2011).

<u>N2-HW introgression lines</u>

CX12004 (*kyIR54*), CX11561 (*kyIR26*), CX11562 (*kyIR32*), CX11564 (*kyIR27*), CX11879 (*kyIR38*), CX11881 (*kyIR40*), CX11900 (*kyIR42*), CX11901 (*kyIR28*), CX11905 (*kyIR46*), CX11930 (*kyIR48*), CX11931(*kyIR49*), CX11932 (*kyIR50*), CX11933 (*kyIR51*), CX11934 (*kyIR52*), CX12005 (*kyIR55*), CX12006 (*kyIR56*), CX12769 (*kyIR62*), CX12771 (*kyIR64*), CX12773 (*kyIR66*), CX12774 (*kyIR67*), CX12775 (*kyIR68*), CX12776 (*kyIR69*), CX12777 (*kyIR70*), CX12778 *(kyIR71*), CX12780 (*kyIR73*), CX12781 (*kyIR74*), CX12782 (*kyIR75*), CX12783 (*kyIR76,* QTL2, CX12983 *kyIR76* 2X backcrossed to N2), CX12772 (*kyIR65*), CX11563 (*kyIR33*),

<u>N2-HW introgression lines</u> (DOROSZUK *et al.* 2009)

ewIR45, ewIR46, ewIR47, ewIR49, ewIR50, ewIR51, ewIR52, ewIR53 (QTL7, CX12986, *ewIR53* 2X backcrossed to N2)*, ewIR54, ewIR55*, *ewIR56, ewIR58, ewIR60, ewIR61, ewIR62, ewIR64, ewIR65, ewIR66, ewIR67, ewIR68, ewIR69, ewIR70, ewIR71*(QTL9, CX12995*, ewIR71* 2X backcrossed to N2*), ewIR72, ewIR73, ewIR74, ewIR75, ewIR76, ewIR77*

N2 and CB4856 Strain History

Bargmann Lab N2 animals were thawed from a frozen stock of N2 animals from the Horvitz Lab frozen down in 1986. Bargmann CB4856 animals were obtained from the CGC in 2007. These strains were rethawed approximately every 3 months. Starting N2 and CB4856 strains and RIAIL lines were from Kruglyak lab. Strains were obtained and maintained as described in (ROCKMAN and KRUGLYAK 2009). Starting N2 and CB4856 strains and CSS lines were from Man-Wah Tan's lab. Starting N2 and CB4856 strains from Bargmann and Tan labs were tested and found to have non-significantly different *Serratia-E. coli* bacteria choice phenotypes.

References

BARRIERE, A., and M. A. FELIX, 2005 High local genetic diversity and low outcrossing rate in *Caenorhabditis elegans* natural populations. Curr Biol **15:** 1176-1184.

DOROSZUK, A., L. B. SNOEK, E. FRADIN, J. RIKSEN and J. KAMMENGA, 2009 A genome-wide library of CB4856/N2 introgression lines of *Caenorhabditis elegans*. Nucleic Acids Res **37:** e110.

FATT, H. V., and E. C. DOUGHERTY, 1963 Genetic control of differential heat tolerance in two strains of the nematode *Caenorhabditis elegans*. Science **141:** 266-267.

GLAUSER, D. A., W. C. CHEN, R. AGIN, B. L. MACINNIS, A. B. HELLMAN *et al.*, 2011 Heat avoidance is regulated by transient receptor potential (TRP) channels and a neuropeptide signaling pathway in *Caenorhabditis elegans*. Genetics **188:** 91-103.

HABER, M., M. SCHUNGEL, A. PUTZ, S. MULLER, B. HASERT *et al.*, 2005 Evolutionary history of *Caenorhabditis elegans* inferred from microsatellites: evidence for spatial and temporal genetic differentiation and the occurrence of outbreeding. Mol Biol Evol **22:** 160-173.

HODGKIN, J., and T. DONIACH, 1997 Natural variation and copulatory plug formation in *Caenorhabditis elegans*. Genetics **146:** 149-164.

ROCKMAN, M. V., and L. KRUGLYAK, 2009 Recombinational landscape and population genomics of *Caenorhabditis elegans*. PLoS Genet **5:** e1000419

File S2

Movie of N2 worms in bacterial choice assay

File S2 is available for download at http://www.g3journal.org/lookup/suppl/doi:10.1534/g3.113.008649/-/DC1.

Files S3-S5

Phenotypes and Genotypes of RIAILs and Introgression Lines

Available for download at http://www.g3journal.org/lookup/suppl/doi:10.1534/g3.113.008649/-/DC1.

File S3 RIAIL bacterial choice phenotypes
File S4 Chromosome IV Introgression lines, bacterial choice phenotypes and genotypes
File S5 Chromosome V Introgression lines, bacterial choice phenotypes and genotypes

File S6

Additional explanation of Chromosome IV QTLs determined by common segment method.

QTLs in bold are supported by more than one introgression line.

**Introgression lines**	**QTL (Mb)**	**NILs that define QTLs**
**First set of *kyIR* lines**	**QTL1** (~2.29 - ~4.99)	Common HW sequence present in HW-phenotype strains (*kyIR28, kyIR73, kyIR26, kyIR51, kyIR52 and kyIR27).* Likely also suppressor(s) in this region, but each would only be defined by one line.
**Nested *kyIR* lines**	**QTL2** (~0.79 - ~1.03)	Common HW sequence present in HW-phenotype strains (*kyIR76* and *kyIR74*) and absent in N2-phenotype strain *(kyIR54).*
	**QTL3** (~1.80 - ~2.76)	Common HW sequence present in HW-phenotype strains (*kyIR67*, *kyIR68*, *kyIR75*, and *kyIR65)* and absent in N2-phenotype strains (*kyIR66, kyIR42, and kyIR71)*.
	QTL4 (~3.92 - ~4.99)	HW sequence present in HW-phenotype strain *kyIR28* and absent in N2-phenotype strain *kyIR62*. Only defined by one line.
	**Suppressor QTL5** (~1.04 -~1.22)	Common HW sequence present in N2-phenotype strains that contain QTL2 (*kyIR69*, *kyIR66*, *kyIR42*, *kyIR71*).
	Suppressor QTL6 (~2.76 -~4.99)	HW sequence present in N2-phenotype strain that contains QTL3 (*kyIR62*). Only defined by one line.
***ewIR* lines**	**QTL7** (~2.76 - ~3.35)	Common HW sequence present in HW-phenotype strains (*ewIR50* and *ewIR53*)
	**QTL8** (~9.10 - ~13.68)	Common HW sequence present in HW-phenotype strains (*ewIR55* and *ewIR54*).
**All lines**	**QTL2** (~0.79 - ~1.03)	Common HW sequence present in HW-phenotype strains (*kyIR76* and *kyIR74*) and absent in N2-phenotype strain *(kyIR54).*
	**QTL3** (~2.29 - ~2.76)	Common HW sequence present in HW-phenotype strains (*kyIR67*, *kyIR68*, *kyIR75*, and *kyIR65)* and absent in N2-phenotype strains (*ewIR45* and *ewIR46*).
	QTL4 (~3.92 - ~4.99)	HW sequence present in HW-phenotype strain *kyIR28* and absent in N2-phenotype strains *kyIR62 and ewIR47*. Only defined by one HW-phenotype strain.
	**Suppressor QTL5** (~1.04 -~1.22)	Common HW sequence present in N2-phenotype strains that contain QTL2 (*kyIR69*, *kyIR66*, *kyIR42*, *kyIR71*, *ewIR45* and *ewIR46*).
	**Suppressor QTL6** (~2.76 -~3.92)	Common HW sequence present in N2-phenotype strains that contain QTL3 (*ewIR47* and *kyIR62*).
	**QTL7** (~2.76 - ~3.35)	Common HW sequence present in HW-phenotype strains (*ewIR53* and *kyIR73*)
	QTL8 (~9.10 - ~13.68)	HW sequence present in HW-phenotype strains *ewIR55*. Strain *ewIR54* not significant when analyzed with all lines, but is when analyzed with only *ewIR* lines.

File S7

Explanation of Chromosome IV QTLs defined by sequential minimum spanning tree method.
Lists introgression lines that define each QTL by sequential MST method.

**Introgression lines**	**QTL (Mb)**	**NILs that define QTLs**
**First set of *kyIR* lines**	None	No significant differences detected.
**Nested *kyIR* lines**	QTL m2 (~0.79 - ~1.03)	HW sequence present in HW-phenotype strain *kyIR76* and absent in N2-phenotype strain *kyIR54.*
***ewIR* lines**	QTL m7 (~2.76 - ~3.35)	HW sequence present in HW-phenotype strain *ewIR53* and absent in N2.
	QTL m8 (~10.12 - ~12.75)	Defined by HW sequence present in HW phenotype strain *ewIR58* and absent in N2-phenotype strain *ewIR60*
**All lines**	QTL m2 (~0.79 - ~1.03)	HW sequence present in HW-phenotype strain *kyIR76* and absent in N2-phenotype strain *kyIR54*.
	QTL m7 (~2.76 - ~3.35)	HW sequence present in HW-phenotype strain *ewIR53* and absent in N2.
	Suppressor QTL m6 (~2.76 - ~3.92 Mb).	Additional HW sequence present in N2-phenotype strain *ewIR47* and absent in HW-phenotype strain *kyIR65*.

File S8

Explanation of Chromosome V QTLs by common segment and sequential minimum spanning tree methods.

Lists introgression lines that define each QTL by common segment and sequential MST method.

**Method**	**QTL (Mb)**	**NILs that define QTLs**
Common segment method	QTL9 (~10.91 – ~13.95)	Common HW sequence present in HW-phenotype strains (*ewIR69* and *ewIR71*). All other tested strains have N2-like phenotype.
Common segment method	Suppressor QTL10 (~10.38 - ~end)	Strain *ewIR70* contains QTL9, but has an N2-like phenotype.
Sequential MST method	QTL m9 (~4.55 – ~10.91)	Strain (*ewIR69*) has a significantly more HW-like phenotype than strain *(kyIR70).* QTL m9 is defined by the additional HW-sequence present in strain *ewIR69* and absent in *ewIR70.*

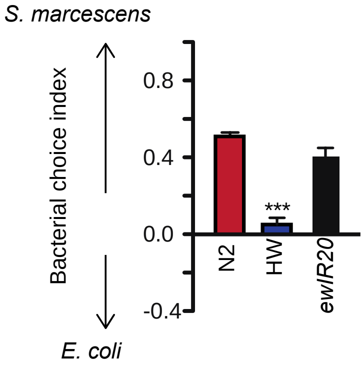

Figure S1 Bacterial choice behavior of introgression strain with chromosome II QTL predicted by RIAIL analysis. Bacterial choice index of N2, HW and *ewIR20* (II ~0 - ~3.4 Mb, CB4856 >N2), which contains the chromosome II sequence near 2,808,858 predicted to contain a HW QTL by RIAILS (Figure 2). ***p < 0.001 compared to N2 by ANOVA with Dunnett test, n $\geq$ 5 assays. Error bars represent S.E.M. The strain *ewIR20* may have an N2-like phenotype because this region does not contain a QTL or because this region must interact epistatically with other QTLs to generate a HW-like phenotype. Note that control values for N2 and HW in this set of experiments are slightly different from those in Figure 1.

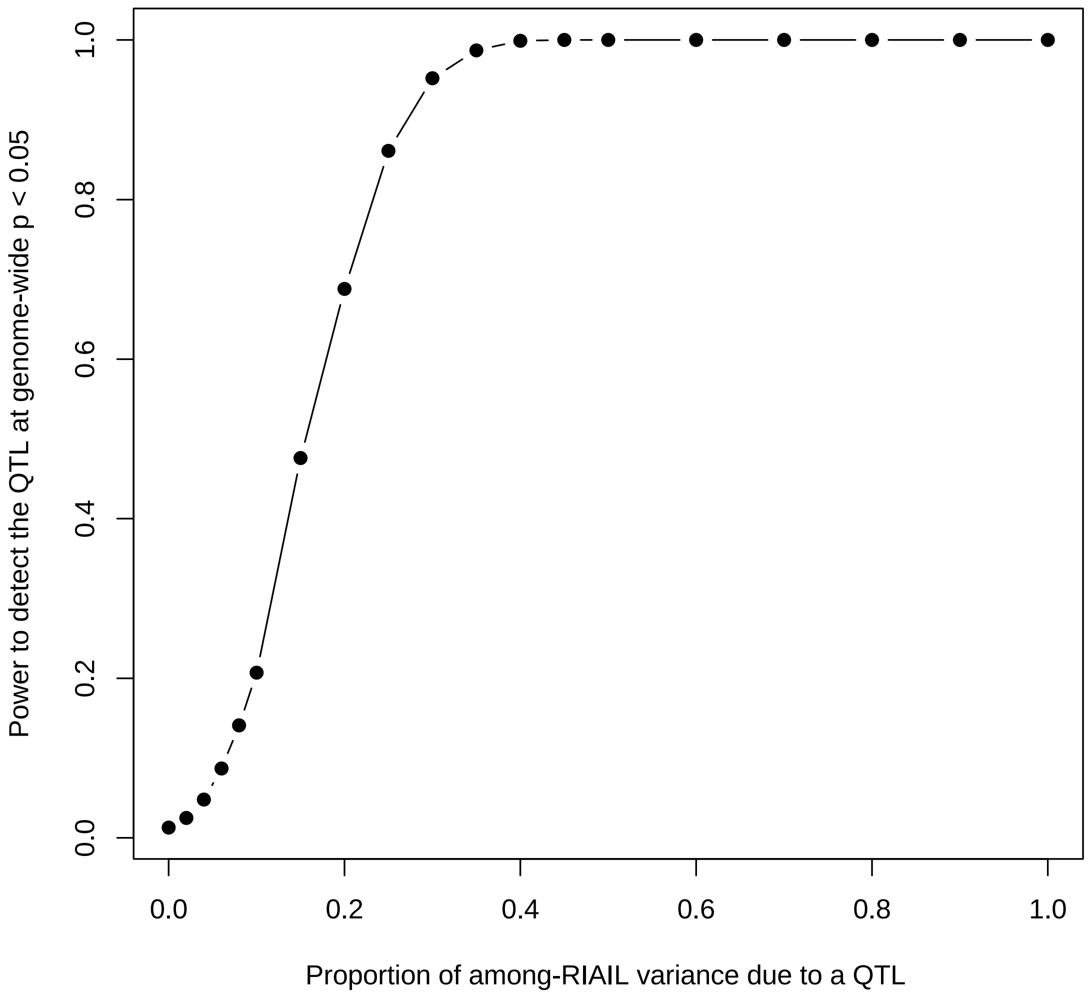

Figure S2 Power analysis of RIAILs. Power to detect QTLs on chromosomes IV and V in the RIAIL panel. For each QTL effect size (measured in terms of its proportion of among-RIAIL variance explained), we simulated 1000 datasets with a QTL on chromosome IV. The fraction of the 1000 datasets in which we were then able to detect a QTL on chromosome IV is an estimate of our power. To account for allele frequency variation along the chromosomes, we repeated the power analysis for QTLs at markers that span the range of minor allele frequencies on chromosome IV and chromosome V and observed negligible effects on power.
