# Supplementary material for: Multigenic Natural Variation Underlies Caenorhabditis elegans Olfactory Preference for the Bacterial Pathogen Serratia marcescens
Source: G3 (Bethesda). 2013 Dec 17;4(2):265–76. doi: 10.1534/g3.113.008649 (PMC3931561; doi:10.1534/g3.113.008649)
Supplement: Supporting Information [file supp_g3.113.008649_FigureS1.pdf]

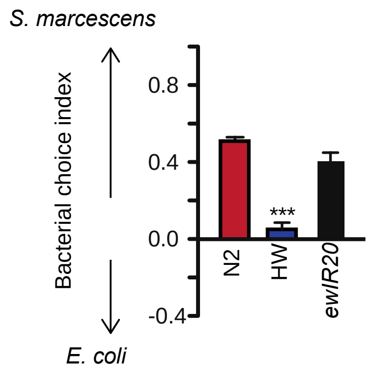


**Figure S1 Bacterial choice behavior of introgression strain with chromosome II QTL predicted by RIAIL analysis.** Bacterial choice index of N2, HW and *ewIR20* (II ~0 - ~3.4 Mb, CB4856 >N2), which contains the chromosome II sequence near 2,808,858 predicted to contain a HW QTL by RIAILS (Figure 2). ***$p < 0.001$ compared to N2 by ANOVA with Dunnett test, n $\geq$ 5 assays. Error bars represent S.E.M. The strain *ewIR20* may have an N2-like phenotype because this region does not contain a QTL or because this region must interact epistatically with other QTLs to generate a HW-like phenotype. Note that control values for N2 and HW in this set of experiments are slightly different from those in Figure 1.
