# Supplementary material for: Multigenic Natural Variation Underlies Caenorhabditis elegans Olfactory Preference for the Bacterial Pathogen Serratia marcescens
Source: G3 (Bethesda). 2013 Dec 17;4(2):265–76. doi: 10.1534/g3.113.008649 (PMC3931561; doi:10.1534/g3.113.008649)
Supplement: Supporting Information [file supp_g3.113.008649_FigureS2.pdf]

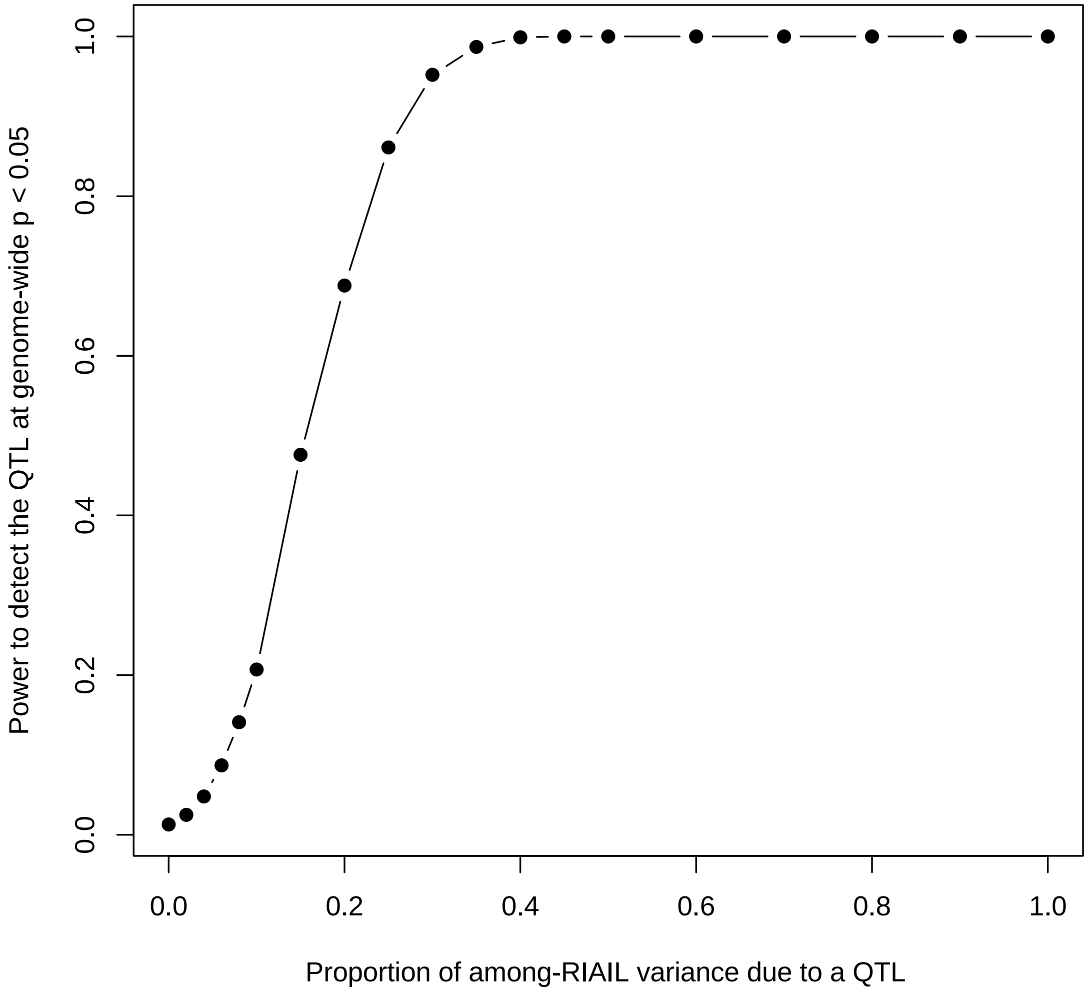

Figure S2 Power analysis of RIAILs. Power to detect QTLs on chromosomes IV and V in the RIAIL panel. For each QTL effect size (measured in terms of its proportion of among-RIAIL variance explained), we simulated 1000 datasets with a QTL on chromosome IV. The fraction of the 1000 datasets in which we were then able to detect a QTL on chromosome IV is an estimate of our power. To account for allele frequency variation along the chromosomes, we repeated the power analysis for QTLs at markers that span the range of minor allele frequencies on chromosome IV and chromosome V and observed negligible effects on power.
