# Supplementary material for: Multigenic Natural Variation Underlies Caenorhabditis elegans Olfactory Preference for the Bacterial Pathogen Serratia marcescens
Source: G3 (Bethesda). 2013 Dec 17;4(2):265–76. doi: 10.1534/g3.113.008649 (PMC3931561; doi:10.1534/g3.113.008649)
Supplement: Supporting Information [file supp_g3.113.008649_FileS1.pdf]

File S1

Supplemental Methods

C. elegans **Strains**

'Wild-type' strains

Strain	*Origin*
N2	Bristol, England (FATT and DOUGHERTY 1963)
CB4856	Hawaii, USA (described in (HODGKIN and DONIACH 1997))
MY1	Lingen, Germany (HABER *et al.* 2005)
MY14	Mecklenbeck, Germany (HABER *et al.* 2005)
JU258	Madeira, Portugal (BARRIERE and FELIX 2005)

Chromosome substitution strains

WE5236 (CSSI), WE5237 (CSSII), WE5238 (CSSIII), WE5239 (CSSIV), WE5240 (CSSV), WE5241 (CSSX) (GLAUSER *et al.* 2011).

N2-HW introgression lines

CX12004 (*kyIR54*), CX11561 (*kyIR26*), CX11562 (*kyIR32*), CX11564 (*kyIR27*), CX11879 (*kyIR38*), CX11881 (*kyIR40*), CX11900 (*kyIR42*), CX11901 (*kyIR28*), CX11905 (*kyIR46*), CX11930 (*kyIR48*), CX11931(*kyIR49*), CX11932 (*kyIR50*), CX11933 (*kyIR51*), CX11934 (*kyIR52*), CX12005 (*kyIR55*), CX12006 (*kyIR56*), CX12769 (*kyIR62*), CX12771 (*kyIR64*), CX12773 (*kyIR66*), CX12774 (*kyIR67*), CX12775 (*kyIR68*), CX12776 (*kyIR69*), CX12777 (*kyIR70*), CX12778 (*kyIR71*), CX12780 (*kyIR73*), CX12781 (*kyIR74*), CX12782 (*kyIR75*), CX12783 (*kyIR76,* QTL2, CX12983 *kyIR76* 2X backcrossed to N2), CX12772 (*kyIR65*), CX11563 (*kyIR33*),

N2-HW introgression lines (DOROSZUK *et al.* 2009)

ewIR45, ewIR46, ewIR47, ewIR49, ewIR50, ewIR51, ewIR52, ewIR53 (QTL7, CX12986, *ewIR53* 2X backcrossed to N2), *ewIR54, ewIR55*, *ewIR56, ewIR58, ewIR60, ewIR61, ewIR62, ewIR64, ewIR65, ewIR66, ewIR67, ewIR68, ewIR69, ewIR70, ewIR71*(QTL9, CX12995*, ewIR71* 2X backcrossed to N2*), ewIR72, ewIR73, ewIR74, ewIR75, ewIR76, ewIR77*

N2 and CB4856 Strain History

Bargmann Lab N2 animals were thawed from a frozen stock of N2 animals from the Horvitz Lab frozen down in 1986. Bargmann CB4856 animals were obtained from the CGC in 2007. These strains were rethawed approximately every 3 months. Starting N2 and CB4856 strains and RIAIL lines were from Kruglyak lab. Strains were obtained and maintained as described in (ROCKMAN and KRUGLYAK 2009). Starting N2 and CB4856 strains and CSS lines were from Man-Wah Tan's lab. Starting N2 and CB4856 strains from Bargmann and Tan labs were tested and found to have non-significantly different *Serratia-E. coli* bacteria choice phenotypes.

References

BARRIERE, A., and M. A. FELIX, 2005 High local genetic diversity and low outcrossing rate in *Caenorhabditis elegans* natural populations. Curr Biol **15:** 1176-1184.

DOROSZUK, A., L. B. SNOEK, E. FRADIN, J. RIKSEN and J. KAMMENGA, 2009 A genome-wide library of CB4856/N2 introgression lines of *Caenorhabditis elegans*. Nucleic Acids Res **37:** e110.

FATT, H. V., and E. C. DOUGHERTY, 1963 Genetic control of differential heat tolerance in two strains of the nematode *Caenorhabditis elegans*. Science **141:** 266-267.

GLAUSER, D. A., W. C. CHEN, R. AGIN, B. L. MACINNIS, A. B. HELLMAN *et al.*, 2011 Heat avoidance is regulated by transient receptor potential (TRP) channels and a neuropeptide signaling pathway in *Caenorhabditis elegans*. Genetics **188:** 91-103.

HABER, M., M. SCHUNGEL, A. PUTZ, S. MULLER, B. HASERT *et al.*, 2005 Evolutionary history of *Caenorhabditis elegans* inferred from microsatellites: evidence for spatial and temporal genetic differentiation and the occurrence of outbreeding. Mol Biol Evol **22:** 160-173.

HODGKIN, J., and T. DONIACH, 1997 Natural variation and copulatory plug formation in *Caenorhabditis elegans*. Genetics **146:** 149-164.

ROCKMAN, M. V., and L. KRUGLYAK, 2009 Recombinational landscape and population genomics of *Caenorhabditis elegans*. PLoS Genet **5:** e1000419
