# Supplementary material for: Multigenic Natural Variation Underlies Caenorhabditis elegans Olfactory Preference for the Bacterial Pathogen Serratia marcescens
Source: G3 (Bethesda). 2013 Dec 17;4(2):265–76. doi: 10.1534/g3.113.008649 (PMC3931561; doi:10.1534/g3.113.008649)
Supplement: Supporting Information [file supp_g3.113.008649_FileS6.pdf]

File S6

Additional explanation of Chromosome IV QTLs determined by common segment method.
QTLs in bold are supported by more than one introgression line.

**Introgression lines**	**QTL (Mb)**	**NILs that define QTLs**
**First set of *kyIR* lines**	**QTL1** (~2.29 - ~4.99)	Common HW sequence present in HW-phenotype strains (*kyIR28, kyIR73, kyIR26, kyIR51, kyIR52 and kyIR27).* Likely also suppressor(s) in this region, but each would only be defined by one line.
**Nested *kyIR* lines**	**QTL2** (~0.79 - ~1.03)	Common HW sequence present in HW-phenotype strains (*kyIR76* and *kyIR74*) and absent in N2-phenotype strain *(kyIR54).*
	**QTL3** (~1.80 - ~2.76)	Common HW sequence present in HW-phenotype strains (*kyIR67*, *kyIR68*, *kyIR75*, and *kyIR65)* and absent in N2-phenotype strains (*kyIR66, kyIR42, and kyIR71)*.
	QTL4 (~3.92 - ~4.99)	HW sequence present in HW-phenotype strain *kyIR28* and absent in N2-phenotype strain *kyIR62*. Only defined by one line.
	**Suppressor QTL5** (~1.04 -~1.22)	Common HW sequence present in N2-phenotype strains that contain QTL2 (*kyIR69*, *kyIR66*, *kyIR42*, *kyIR71*).
	Suppressor QTL6 (~2.76 -~4.99)	HW sequence present in N2-phenotype strain that contains QTL3 (*kyIR62*). Only defined by one line.
***ewIR* lines**	**QTL7** (~2.76 - ~3.35)	Common HW sequence present in HW-phenotype strains (*ewIR50* and *ewIR53*)
	**QTL8** (~9.10 - ~13.68)	Common HW sequence present in HW-phenotype strains (*ewIR55* and *ewIR54*).
**All lines**	**QTL2** (~0.79 - ~1.03)	Common HW sequence present in HW-phenotype strains (*kyIR76* and *kyIR74*) and absent in N2-phenotype strain *(kyIR54).*
	**QTL3** (~2.29 - ~2.76)	Common HW sequence present in HW-phenotype strains (*kyIR67*, *kyIR68*, *kyIR75*, and *kyIR65)* and absent in N2-phenotype strains (*ewIR45* and *ewIR46*).
	QTL4 (~3.92 - ~4.99)	HW sequence present in HW-phenotype strain *kyIR28* and absent in N2-phenotype strains *kyIR62 and ewIR47*. Only defined by one HW-phenotype strain.
	**Suppressor QTL5** (~1.04 -~1.22)	Common HW sequence present in N2-phenotype strains that contain QTL2 (*kyIR69*, *kyIR66*, *kyIR42*, *kyIR71*, *ewIR45* and *ewIR46*).
	**Suppressor QTL6** (~2.76 -~3.92)	Common HW sequence present in N2-phenotype strains that contain QTL3 (*ewIR47* and *kyIR62*).
	**QTL7** (~2.76 - ~3.35)	Common HW sequence present in HW-phenotype strains (*ewIR53* and *kyIR73*)
	QTL8 (~9.10 - ~13.68)	HW sequence present in HW-phenotype strains *ewIR55*. Strain *ewIR54* not significant when analyzed with all lines, but is when analyzed with only *ewIR* lines.
