# Supplementary material for: Multigenic Natural Variation Underlies Caenorhabditis elegans Olfactory Preference for the Bacterial Pathogen Serratia marcescens
Source: G3 (Bethesda). 2013 Dec 17;4(2):265–76. doi: 10.1534/g3.113.008649 (PMC3931561; doi:10.1534/g3.113.008649)
Supplement: Supporting Information [file supp_g3.113.008649_FileS7.pdf]

File S7

Explanation of Chromosome IV QTLs defined by sequential minimum spanning tree method.
Lists introgression lines that define each QTL by sequential MST method.

**Introgression lines**	**QTL (Mb)**	**NILs that define QTLs**
**First set of *kyIR* lines**	None	No significant differences detected.
**Nested *kyIR* lines**	QTL m2 (~0.79 - ~1.03)	HW sequence present in HW-phenotype strain *kyIR76* and absent in N2-phenotype strain *kyIR54.*
***ewIR* lines**	QTL m7 (~2.76 - ~3.35)	HW sequence present in HW-phenotype strain *ewIR53* and absent in N2.
	QTL m8 (~10.12 - ~12.75)	Defined by HW sequence present in HW phenotype strain *ewIR58* and absent in N2-phenotype strain *ewIR60*
**All lines**	QTL m2 (~0.79 - ~1.03)	HW sequence present in HW-phenotype strain *kyIR76* and absent in N2-phenotype strain *kyIR54*.
	QTL m7 (~2.76 - ~3.35)	HW sequence present in HW-phenotype strain *ewIR53* and absent in N2.
	Suppressor QTL m6 (~2.76 - ~3.92 Mb).	Additional HW sequence present in N2-phenotype strain *ewIR47* and absent in HW-phenotype strain *kyIR65*.
