# Supplementary material for: Multigenic Natural Variation Underlies Caenorhabditis elegans Olfactory Preference for the Bacterial Pathogen Serratia marcescens
Source: G3 (Bethesda). 2013 Dec 17;4(2):265–76. doi: 10.1534/g3.113.008649 (PMC3931561; doi:10.1534/g3.113.008649)
Supplement: Supporting Information [file supp_g3.113.008649_FileS8.pdf]

File S8

Explanation of Chromosome V QTLs by common segment and sequential minimum spanning tree methods.

Lists introgression lines that define each QTL by common segment and sequential MST method.

**Method**	**QTL (Mb)**	**NILs that define QTLs**
Common segment method	QTL9 (~10.91 – ~13.95)	Common HW sequence present in HW-phenotype strains (*ewIR69* and *ewIR71*). All other tested strains have N2-like phenotype.
Common segment method	Suppressor QTL10 (~10.38 - ~end)	Strain *ewIR70* contains QTL9, but has an N2-like phenotype.
Sequential MST method	QTL m9 (~4.55 – ~10.91)	Strain (*ewIR69*) has a significantly more HW-like phenotype than strain *(kyIR70).* QTL m9 is defined by the additional HW-sequence present in strain *ewIR69* and absent in *ewIR70.*
